# Non-ST elevation acute coronary syndrome in women and the elderly: recent updates and stones still left unturned

**DOI:** 10.12688/f1000research.16492.1

**Published:** 2018-11-29

**Authors:** Tina Varghese, Nanette K. Wenger

**Affiliations:** 1 Department of Medicine, Division of Cardiology, Emory University School of Medicine, Atlanta, GA, USA; 2 Emory Heart and Vascular Center, Emory Women's Heart Center, Atlanta, GA, USA

**Keywords:** non-ST elevation myocardial infarction, acute coronary syndrome, women, elderly

## Abstract

Despite the growing awareness of adverse events with acute coronary syndrome and vigilance to refine pharmacological and interventional therapies, the understanding of how these events present in and affect women and the elderly remains limited. Pathophysiological differences in these subgroups and under-representation in large trials create a medical gap in sex- and age-related outcomes and in our knowledge of how best to detect, diagnose, and treat acute coronary syndrome. This review provides a general overview of recent advances in non-ST elevation myocardial infarction management in women and the elderly and elucidates areas where further exploration is needed.

## Introduction

Each year in the US, over 780,000 people experience an acute coronary syndrome (ACS), which in 70% of these patients is non-ST elevation myocardial infarction (NSTEMI) ^1^. In recent decades, our understanding of the pathophysiology, diagnosis, and management of NSTEMI has significantly expanded. However, data for certain subsets of patients—namely women, who represent half of the worldwide population, and the elderly, a group expected to grow from approximately 524 million in 2010 to 1.5 billion in 2050—are less well elucidated ^2^. Both women and the elderly (defined as either older than 65 or older than 75) with NSTEMI are less likely than their male counterparts and younger patients to receive guideline-determined medical therapy (GDMT) and intervention ^3, 4^. One evident contributor to this disparity is the predominance of middle-aged men in randomized controlled trials (RCTs) of cardiovascular disease (CVD), and women and the elderly are significantly under-represented. Women represent 43% and individuals older than 75 represent 37% of myocardial infarction (MI) patients in the US. However, women and the elderly are enrolled in RCTs at rates of 25% and 9%, respectively ^5^; therefore, risk–benefit ratios for available therapies rely heavily on data extrapolation. Currently, the 5-year risk of death from NSTEMI for women is 42% (versus 29% in men) and 1-year mortality risk after NSTEMI is 20% for patients at least 75 years old and 25% in those older than 85 ^6, 7^. In contrast, the 30-day and 31- to 365-day mortality risk after any MI (STEMI and NSTEMI) for patients under 50 years of age is about 3.2% and 1.6%, respectively, suggesting a 1-year mortality risk of less than 5% in this younger population ^8^.

Increased efforts to intensify inclusivity of these populations in RCTs and apply GDMT for all patients without contraindications are likely responsible for the recently improved 30-day and 1-year mortality outcomes in women and the elderly with NSTEMI ^9– 11^. This review examines contemporary updates in the management of women and elderly patients with NSTEMI and highlights areas of uncertainty that require further exploration.

## Pathophysiology

The typical pathophysiology underlying ACS is the subtotal, thrombotic occlusion of an epicardial coronary artery with resulting myocardial oxygen supply-and-demand mismatch. Plaque rupture causes half of coronary thrombosis events, and plaque erosion and vasoconstriction at the location of the culprit plaque are responsible for MIs with intact fibrous caps ^12^.

### Elderly

Special considerations in the elderly which predispose this group to a higher incidence and more ominous prognosis of atherothrombotic disease are the presence of increased comorbidities, greater complexity of coronary artery lesions, and the direct effects of aging on the heart. Such detrimental age-related effects include reduced vessel elasticity, attenuated atheroprotective effects of high-density lipoproteins, impaired regenerative ability of cells, endothelial dysfunction, increased tendency for coagulation, and pro-inflammatory state ^13^. For instance, elevated C-reactive protein and interleukin-6 levels are reported in the elderly and result in an amplified immune response from substantial release of pro-inflammatory cytokines; this phenomenon alters normal fibrinolysis pathways, contributing to both the high occurrence of MI in this population and a worse prognosis afterwards. Additionally, the age-related deterioration in organ capacities can negatively impact pharmacodynamics and pharmacokinetics, which may result in undesired medication interactions and side effects ^13^.

### Women

In addition to traditional CVD risk factors, there are additional pathophysiological processes experienced by women that require elucidation for appropriate diagnostic and therapeutic protocols to be established. Such areas of ambiguity include the effects of reproductive hormones on inflammatory markers, fat distribution, and atherosclerotic burden as well as a clearer understanding of mental stress-induced and vascular dysfunction-induced ischemia, which is believed to occur more commonly in women than in men and demonstrates the complexity of ACS beyond simply culprit lesion diagnosis ^14– 16^. Microvascular dysfunction—theorized to develop from a sex-specific remodeling response to arterial injury or atherosclerosis—accounts for why women have less anatomical coronary artery disease (CAD) but paradoxically more angina and ischemia than do men ^17^.

In the PESA (Progression of Early Subclinical Atherosclerosis) study of asymptomatic middle-aged patients, the prevalences of subclinical atherosclerotic heart disease in men versus women were 15% and 3% (40 to 44 years of age; *p* <0.001), 24% and 5% (45 to 49 years of age; *p* <0.001), and 43% and 10% (50 to 54 years of age; *p* <0.001), respectively ^18^. Although multiple factors contribute to the presence of CAD, estrogen is believed to play a protective and pleiotropic role against CAD development in premenopausal women and may partially explain the aforementioned and widening disparity in heart disease prevalence with age between the sexes. Estrogen reduces platelet reactivity and thus helps inhibit platelet aggregation in premenopausal women because of the presence of estrogen receptors on platelet surfaces ^19^. Estrogen also improves traditional CVD risk factors, improving lipid levels and reducing the incidence of type 2 diabetes ^20^. Aside from microvascular and hormonal influences, non-traditional risk factors account for sex differences in CAD, including psychosocial risk factors (for example, low socioeconomic status, anxiety and depression, and social isolation), systemic autoimmune disease, and complications of pregnancy (for example, hypertension- and diabetes-related and preterm delivery) ^17, 21, 22^.

Whereas plaque *rupture* is the major pathophysiological cause (76%) of thrombotic, fatal coronary artery occlusions in men, it accounts for only 55% of fatal MIs in women ^23^. Higher occurrences of plaque *erosion* are noted in women, especially younger women ^14, 24^. Plaque erosion and the less-common non-atherosclerotic mechanisms for MI, such as coronary artery spasm and spontaneous coronary artery dissection, help explain the more frequent finding of ACS without angiographically obstructive disease in women compared with men ^25, 26^. The 2018 publication of the Fourth Universal Definition of MI clarifies the existence of MINOCA (myocardial infarction with non-obstructive coronary arteries), which implies the presence of ischemia-induced myocyte injury without obstructive CAD (at least 50% diameter stenosis in a major epicardial artery) as its etiology. MINOCA is more commonly seen in women than in men and in NSTEMI than in STEMI ^26^. This broadened nomenclature for MI classification creates opportunities to improve methods for differentiating plaque characteristics, discovering more specific management approaches for each underlying mechanism of ACS, and improving outcomes.

## Presentation

Although both women and elderly patients usually present with typical symptoms of MI, atypical presentations (for example, back pain, nausea, dyspnea, acute fatigue, and lack of pain) are more common in these subgroups than in men ^27^.

### Elderly

**Figure 1.**
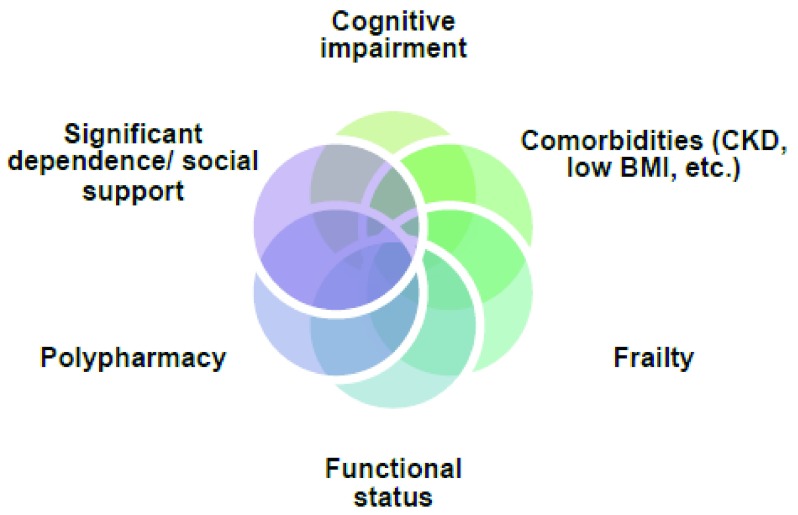
Unique factors to consider in risk stratification of elderly patients presenting with acute coronary syndrome. BMI, body mass index; CKD, chronic kidney disease

With increasing age, the number of patients with ACS who present with Killip class III or IV congestive heart failure symptoms rises ^28^. Also notable in the elderly is the occurrence of abnormal baseline electrocardiograms, which can obscure the findings of ischemia. Such variability in clinical and electrocardiographic presentation in the elderly can cause ACS misdiagnosis and requires a higher level of suspicion of a coronary event. Moreover, frailty, polypharmacy, and features unique to the elderly ( Figure 1) make risk stratification, treatment, and outcomes a further challenge but are vital for appropriately tailored management.

### Women

The VIRGO (Variation in Recovery: Role of Gender on Outcomes of Young AMI [Acute MI] Patients) study, in which nearly 3,000 people were interviewed during their index MI hospitalization, demonstrated that women are more likely to present with non-chest pain symptoms and are more likely to ascribe their MI symptoms to anxiety or stress ^29^. Younger women (younger than 45) with MI were much more likely than age-matched men to present without chest pain; the lack of chest pain is associated with higher mortality rates, although this correlation disappears with age ^30^. The higher death rates in young women possibly result from delayed diagnosis and perhaps also from differing pathophysiology (for example, younger women experience more plaque erosion than rupture) that may not be appropriately addressed with current interventional strategies.

## Pharmacology

The medical management of ACS uses anti-ischemic medications (for example, beta-blockers and nitrates), secondary prevention medications (for example, statins), medications to help improve systolic dysfunction if present (for example, beta-blockers and angiotensin-converting enzyme [ACE] inhibitors), and antithrombotic therapy ^1^. Special attention should be paid to polypharmacy, age-related change in pharmacokinetics, and drug–drug interactions in the older population and teratogenic consequences in pregnant or childbearing-aged women.

### Elderly

Advanced age is associated with excess dosing of antithrombotic medications in NSTEMI ^31^. Weight and renal clearance/glomerular filtration rate should be considered when dosing antiplatelet and anticoagulant therapies ^1^. Hemorrhagic complications of ACS management are particularly feared in the elderly because of the increased susceptibility to bleeding and risk of significant sequelae from such events. For patients requiring both dual antiplatelet therapy (DAPT) for NSTEMI and anticoagulation with either warfarin or a direct oral anticoagulant (DOAC) for other conditions (that is, atrial fibrillation, prosthetic valves, or left ventricular thrombus), the American College of Cardiology/American Heart Association (ACC/AHA) does not have specific recommendations for which P2Y12 inhibitor should be used in this scenario. However, newer antiplatelet agents (for example, ticagrelor) may want to be avoided in patients requiring triple therapy owing to their increased bleeding risk without easy reversibility ^1^. One of the newer agents, prasugrel, should be avoided in general in those patients who are older than 75, weigh less than 60 kg, or have a history of stroke owing to excessive bleeding risk without clear benefit ^1^.

The 2016 ACC/AHA focused update suggests that in patients with high risk of bleeding (for example, taking an oral anticoagulant medication) or high risk of bleeding complication, discontinuation of P2Y12 medication at 6 months after NSTEMI treated with drug-eluting stent(s) could be considered (class of recommendation IIb and level of evidence C-LD) ^32^. Other considerations to reduce bleeding include the addition of proton pump inhibitors for those at risk of gastrointestinal bleeding while on DAPT, narrowing the international normalized ratio goal to 2.0 to 2.5 if on warfarin, and switching triple therapy to dual earlier on (that is, triple therapy for 1 month post-percutaneous coronary intervention, or post-PCI, followed by DOAC with only clopidogrel or aspirin for 11 months) ^32, 33^. During an NSTEMI, reduced bleeding risk may be achieved by using bivalirudin, a direct thrombin inhibitor, in the elderly instead of glycoprotein IIb/IIIa inhibitor with unfractionated heparin ^1^. Additionally, pooled analysis from three large RCTs of cangrelor, an intravenous platelet inhibitor, administered at the time of PCI showed reduction of the primary composite outcome of death, MI, ischemia-driven revascularization, and stent thrombosis by 19% at 48 hours when compared with clopidogrel, and the benefit was more marked among patients 75 years old or older. An increase in severe bleeding was not observed ^34^. Therefore, this drug represents a potential alternative for patients who require platelet inhibition but cannot tolerate oral agents. Additional studies in the elderly are required before significant further conclusions can be made on benefits versus risks.

### Women

The 2014 ACC/AHA NSTEMI guidelines recommend that NSTEMI in women be treated with the same medications as those used in men ^1^. The one clear exception to routine ACS pharmacological agents in women is in pregnancy; ACE inhibitors, angiotensin receptor blockers, and statins should be avoided because of their teratogenic effects ^35, 36^. After an AMI, menopausal hormone therapy should not be initiated or should be discontinued (if taking already) unless benefits strongly outweigh risks; data from the Women’s Health Initiative RCT and from the Heart and Estrogen/Progestin Replacement Study (HERS) raise concern for increased CV events with estrogen or estrogen–progesterone pills ^37, 38^.

As with the elderly, women with NSTEMI receive excess antithrombotic medication dosing than appropriate for their weight or renal function (or both), and this is partly responsible for the higher risk of in-hospital bleeds and access-related complications after PCI in women ^31^. Women also experience frequent fluctuations in prothrombotic tendency throughout their lives because of menstrual cycles, use of oral contraceptives, pregnancy, menopause, and hormone replacement therapy, requiring careful consideration when using and dosing blood thinners in AMI, including during the prothrombotic state of pregnancy ^19^. Women are less likely than men to be adherent to antiplatelet therapy, and this is possibly because of the higher bleeding rates ^39^. They are likely to experience both underutilization and early discontinuation of other medications categorized as GDMT despite equal efficacy of medications ^40, 41^. In the MINOCA population, patients are less likely to receive beta-blockers, ACE inhibitors, and statins compared with patients with obstructive CAD on 3-month post-angiography follow-up, even though increased mortality and MI rates are associated with this diagnosis; this discrepancy calls for increased evaluation of MINOCA outcomes and awareness of its prognosis despite a more benign anatomical appearance ^42, 43^. Evaluation of new pharmacotherapies should be pursued for improved management of microvascular dysfunction; some evidence suggests benefit from spironolactone, tricyclic antidepressants, and ranolazine ^44– 46^.

## Revascularization

All patients presenting with NSTEMI should receive appropriate pharmacological therapies and undergo evaluation for invasive intervention, which currently consists of two pathways. An invasive strategy refers to coronary angiography, subdivided into early invasive (angiography within 24 hours of NSTEMI presentation) and delayed invasive (within 25 to 72 hours) strategies. An ischemia-guided, previously termed “conservative”, strategy implies coronary angiography for those who (a) have refractory angina despite GDMT as tolerated, (b) demonstrate objective ischemic signs on non-invasive stress imaging, and/or (c) have poor prognostic features: that is, high GRACE (Global Registry of Acute Coronary Events) ( Table 1) or TIMI (Thrombolysis in Myocardial Infarction) risk scores ^1, 47, 48^. A meta-analysis of the FRISC-II, ICTUS, and RITA-3 RCTs showed fewer CV deaths and non-fatal MIs at 5 years with a routine invasive strategy ^49^. Current guidelines recommend immediate coronary angiography in patients with NSTEMI with concomitant refractory angina or electrical/hemodynamic instability and an early invasive strategy for those with high-risk but stable NSTEMI (for example, GRACE risk score of more than 140, change in troponins, or new ST depression on electrocardiogram) ^1^.

**Table 1.** Global Registry of Acute Coronary Events (GRACE) risk score risk factors.

Age in years
Heart rate in beats per minute
Systolic blood pressure in millimeters of mercury
Creatinine in micromoles per liter
Congestive heart failure Killip class
Cardiac arrest at admission
ST segment deviation on electrocardiogram
Elevated cardiac enzymes/markers

Risk assessment tool to predict 6-month risk of mortality or myocardial infarction after initial presentation with acute coronary syndrome.

The recently published VERDICT (Very Early vs. Deferred Invasive evaluation using Computerized Tomography) RCT showed that a very early invasive strategy (<12 hours of diagnosis) did not significantly improve long-term outcomes (median follow-up of 4.3 years) in patients with NSTEMI compared with an invasive strategy performed within 48 to 72 hours of diagnosis, although benefit was noted in a high-risk subgroup of patients with a GRACE risk score of more than 140 ^50^. No benefit was noted in women or patients over the age of 64, but the majority of the studied population were middle-aged men ^50^. Inclusion of women and older patients is needed to definitively assess very early invasive outcomes in these two populations.

### Elderly

Short- and long-term outcome data suggest that an early invasive strategy confers a significant absolute and relative risk reduction in patients 65 years of age or older, yet this population is less likely to receive such treatment because risk factors that put elderly patients at higher risk for morbidity and mortality in ACS may paradoxically be viewed as relative contraindications to invasive management ^6, 51– 53^. However, in patients 75 years old or older, the majority of deaths within 1 year of an NSTEMI originate from cardiac ischemia, and the benefit from revascularization appears to progressively rise with background risk, bolstering the need for better and earlier treatment options ^54, 55^.

A recent meta-analysis demonstrated reduced 30-day and 12-month mortality rates in the invasive compared with the conservative group among an elderly population, but these results were driven primarily by observational studies—which are subject to selection bias—and were no longer evident when only RCTs were analyzed; analysis of the RCTs suggested reduced 1-year re-infarction and possibly reduced stroke rates with the invasive strategy ^56^. Unlike with medical management, the subject of revascularization in older adults contains several recent RCTs dedicated exclusively to this population. The Italian Study, published in 2012, was the first RCT to evaluate revascularization strategies in 313 NSTEMI patients who were 75 or older ^57^. A strong benefit to early revascularization was not observed, and this is likely because the study was underpowered, but it demonstrated a statistically significant reduction in clinical events in patients with elevated troponin levels on admission. Four years later, the After Eighty Study delivered more convincing evidence supporting guideline-recommended routine invasive management of NSTEMI or unstable angina over conservative management in patients aged 80 or older ^58^. The invasive strategy outperformed conservative measures in the composite primary outcome, and the benefit was driven primarily by reductions in MI and urgent revascularizations. Whether the benefit of this strategy existed in patients older than 90 was unclear. Applicability to the elderly population as a whole is perhaps too optimistic, as many older patients were excluded from the study. However, the patients included represent a higher-risk elderly population given the presence of increased comorbidities compared with prior studies, yet fewer contrast-induced nephropathy or major bleeding incidences were noted in the invasive group than in previous trials ^58^. The lower risk of bleeding may be a result of both increased radial artery access (which has been associated with fewer access site complications and bleeding than femoral access) and careful selection of stable patients, further supporting the need for appropriate screening of patients ^58, 59^.

Trials such as the Italian Study and After Eighty Study confirm that invasive management of NSTEMI can and should be safely performed in stable elderly patients despite their clinical complexity. Although such conclusions align with established guidelines, these RCTs are unique in that they are geared toward this specific population, allowing us to forego dependence solely on subgroup analysis and extrapolation. As with PCI evaluation, coronary artery bypass grafting (CABG) evaluation should not be withheld from elderly patients who present with NSTEMI, especially those with diabetes or systolic dysfunction (or both), should their coronary angiography reveal left main or multivessel CAD ^1^.

### Women

A meta-analysis of RCTs involving invasive versus conservative revascularization in patients with NSTEMI between 1970 and 2008 illustrated a 33% reduced odds of MI, death, or re-hospitalization for ACS when using an early invasive strategy in women with high-risk features, such as elevated troponin levels ^60^. An early invasive strategy offered no substantial benefit, and perhaps may cause increased MI or death, in women without biomarker elevation; this is reflected in other studies (for example, TACTICS-TIMI 18) and in current guidelines ^1, 60^. Consistent with European Society of Cardiology guidelines, revascularization in pregnant women has a class IIa recommendation and can be considered if medical therapy is unsuccessful at addressing life-threatening complications ^1, 61^.

Despite the guidelines, women are less likely than men to undergo revascularization with NSTEMI. In a study involving 23,863 patients with ACS, women were less likely than men to receive coronary angiography, revascularization, or CABG both before and after adjustment for confounders ^62^. Causes include inherent gender bias and underestimation of patient risk, atypical symptoms on presentation, conflicting data from post-hoc analysis of trials regarding revascularization benefit, and 1.5- to 4-fold-higher vascular complications from the procedure ^29, 62– 66^. Rahimtoola *et al*. reported a higher unadjusted operative mortality risk and lower long-term survival for women undergoing CABG compared with men, but sex was not an independent risk factor for lower survival ^67^. The BARI (Bypass Angioplasty Revascularization Investigation) trial showed that women did as well as men, if not better, at 5-year follow-up post-CABG and had similar in-hospital mortality rates ^68^. These findings emphasize that careful assessment of risk–benefit ratios must be performed before women undergo PCI or surgical revascularization, but given the clearly established benefit of revascularization in AMI, our medical community must adhere to consistent application of evidence-based approaches and guideline-directed revascularization when appropriate.

## Cardiac rehabilitation

Comprehensive cardiac rehabilitation (CR) is a class 1 recommendation in the continuum of care in patients with AMI, lessening cardiovascular mortality by 26%, reducing hospital admissions by 18%, and improving quality of life ^1, 69– 71^.

### Elderly

Elderly patients tend to be less fit than younger patients at baseline; thus, ACS can accelerate deconditioning. Complications of MIs and of revascularization procedures, compounded with prolonged hospitalizations, lead to further deconditioning ^69^. Therefore, elderly patients stand to gain significant physical and emotional benefit with supervised exercise programs, but they are frequently under-prescribed CR ^72^. Studies have illustrated short-, moderate-, and long-term (3 months, 1 year, and 5 years, respectively) improvement in outcomes in the elderly with CR, and therefore increased awareness of CR availability and a willingness to refer patients should be present ^73, 74^.

### Women

Women are also less likely to be referred to or use CR after an AMI ^72^. Data from the Centers for Disease Control and Prevention show that, among MI survivors, CR is used in only 36.4% of men and in an even lower percentage of women (28.8%) ^75^. A recent study examining the high dropout rate of women found five general explanations: intrapersonal reasons (for example, health beliefs), interpersonal reasons (for example, obligations for work and caregiver of family), logistical reasons (for example, transportation), CR program characteristics (for example, related to CR equipment or timing), and health-system reasons (for example, long waiting list) ^76^. Other barriers to referral or completion of CR (or both) by women include non-modifiable factors (for example, age and diagnosis) and less easily modifiable factors (for example, socioeconomic status/depression and lower education level) ^77^. Methods to improve CR participation must be further assessed and implemented; interventions include more flexible times, incentive programs, and alternative (that is, home-based or smartphone-based) CR models to lessen the burden of barriers to CR completion for all patients with CAD, as this issue extends beyond sex and age ^77^.

## Conclusions

A combination of lack of research outcomes, delay in recognition of ACS symptoms, and less-aggressive interventions because of fear of adverse effects results in a large discrepancy between guideline-emphasized care and the care that is actually provided to women and the elderly. Fortunately, in the past several decades, investigators have begun exploring the distinctive components of heart disease in these two populations to move away from a one-size-fits-all treatment paradigm. “Sex-specific differences” and “geriatric cardiology” are no longer foreign terms but rather arenas of new exploration and spectacular results, but the road to receiving care equal to the care provided to middle-aged men with CAD is challenging. Earlier detection of NSTEMI in these subgroups, awareness of differing pathophysiological mechanisms, increased enrollment in RCTs, evaluation of age- and sex-specific influences on study results, and promotion of secondary prevention by addressing barriers to care are paramount to helping women and the elderly receive guideline-based management in the setting of severe cardiac events. The multiple layers underlying the pathobiological mechanisms behind age- and sex-based differences in CAD epidemiology must be peeled away via basic science and clinical research for improved health outcomes and reduced mortality rates. Only an extensive understanding and appreciation of this intricate medical landscape will allow an all-inclusive ACS treatment approach.

## Abbreviations

ACC/AHA, American College of Cardiology/American Heart Association; ACE, angiotensin-converting enzyme; ACS, acute coronary syndrome; AMI, acute myocardial infarction; CABG, coronary artery bypass grafting; CAD, coronary artery disease; CR, cardiac rehabilitation; CV, cardiovascular; CVD, cardiovascular disease; DAPT, dual antiplatelet therapy; DOAC, direct oral anticoagulant; GDMT, guideline-directed medical therapy; GRACE, Global Registry of Acute Coronary Events; MINOCA, myocardial infarction with non-obstructive coronary arteries; NSTEMI, non-ST segment elevation myocardial infarction; MI, myocardial infarction; PCI, percutaneous coronary intervention; RCT, randomized controlled trial; STEMI, ST segment elevation myocardial infarction; TIMI, Thrombolysis in Myocardial Infarction
